# Peri‐ and Postnatal High Fat Feeding Have Differential Effects on Executive Function and Associated Neurobiology in Aged Male and Female Mice

**DOI:** 10.1111/acel.70223

**Published:** 2025-09-07

**Authors:** Laura Contu, Adam Johnson, Christopher J. Heath, Cheryl A. Hawkes

**Affiliations:** ^1^ Department of Psychology Bristol University Bristol UK; ^2^ Division of Biomedical and Life Sciences Lancaster University Lancaster UK; ^3^ School of Life, Health and Chemical Sciences The Open University Milton Keynes UK

**Keywords:** executive function, lifespan, maternal obesity, mouse, saturated fatty acids

## Abstract

Almost half of pregnant women globally are currently estimated to be overweight or obese. Rates of childhood obesity are also on the rise, in part because of increased consumption of dietary saturated fats. However, the long‐term effect of peri‐ and postnatal high fat (HF) feeding on cognitive function and neuronal expression has not yet been investigated. Male and female C57BL/6J mice born to dams fed a control (C) or high fat (HF) diet were themselves fed either the C or HF diet, generating four experimental groups: C/C, C/HF, HF/C, and HF/HF, representing the peri‐ and postnatal diets, respectively. Offspring underwent evaluation of executive function using the two‐choice paired visual discrimination reversal (PVDR) task at 6 and 12 months of age. Brain tissues were then processed for markers of serotonin, GABA, glutamate, and acetylcholine using RT‐qPCR, Western blotting, and immunohistochemistry. At 6 months of age, C/HF and HF/HF mice performed significantly worse on the PVDR task compared to C/C and HF/C offspring. Perinatal diet did not affect performance at this age. However, 12‐month‐old HF/C males reached criteria more quickly than C/C male mice, suggesting improved cognitive performance. Levels of NeuN were increased in the prefrontal cortex of HF/C animals, alongside a selective increase in markers of acetylcholine. These results suggest that postnatal HF feeding negatively impacts executive function in both adult and aged mice, but consumption of the same diet during the perinatal period only may be beneficial in older age, possibly due to increased cholinergic innervation of the prefrontal cortex.

## Graphical abstract

Postnatal high fat (HF) feeding negatively impacts executive function in both adult and aged mice, but consumption of the same diet during the perinatal period only is beneficial in older age. Maternal HF diet is associated with increased cholinergic innervation of the prefrontal cortex of young, but not aged, offspring.

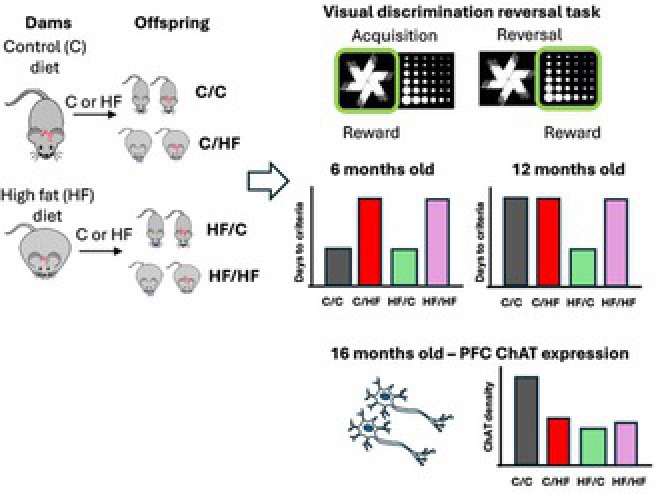

## 1 Introduction

Human neurodevelopment begins by week three of gestation, and brain maturation continues into early adulthood (Kolk and Rakic 2022). Consequently, the brain is sensitive to both the peri‐ and postnatal environment (Tierney and Nelson 3rd 2009). Postnatal consumption of diets that are high in saturated fatty acids (sFA) is associated with cognitive impairment in young and middle‐aged adults and a greater decline in age‐associated memory processing (Francis and Stevenson 2013). In addition, research into the developmental origins of health and disease (DOHaD) also supports a role for maternal obesity and/or early life exposure to a high fat (HF) diet on the long‐term health of the offspring brain. This includes alterations in brain structure, mental health, and cognitive function across childhood, adolescence, and young adulthood (Contu and Hawkes 2017; Hasebe et al. 2021). Recent global estimates suggest that almost half of all pregnant mothers are overweight or obese (Kent et al. 2024) and children raised in Western countries consume elevated amounts of dietary sugar and fat (Warkentin et al. 2025). However, few experimental models have tested the combined effect of peri‐ and postnatal HF consumption on brain function. Moreover, very little is known about the effect of perinatal HF alone or in combination with postnatal HF feeding on cognitive function in aged offspring.

Executive function (EF) is a higher‐order cognitive process that includes measures of planning, organization, attentional shifting, working memory, and behavioral regulation (Diamond 2013). Although most studies support a negative effect of postnatal HF consumption on spatial memory and attentional threshold (Cordner et al. 2019; Evans et al. 2024; Francis and Stevenson 2013), the effect of maternal obesity on measures of EF is unresolved. For example, both higher and lower cognitive performance has been reported in young children whose mothers gained excess weight during pregnancy (Gage et al. 2013; Huang et al. 2014; Tavris and Read 1982). Similarly, animal models have reported that young adult offspring born to mothers fed a HF diet during gestation and/or lactation have both impaired and improved spatial memory (Cordner et al. 2019; Grissom et al. 2015; McKee et al. 2017; Smith et al. 2020). Of the limited studies that have examined aged offspring, work by Di Meco and Pratico found that 18‐month‐old mouse offspring born to dams fed a HF diet during gestation had better spatial memory recall compared to control offspring (Di Meco and Pratico 2019). Interestingly, for reasons that are not fully understood, the impact of maternal obesity on the offspring brain appears to be sexually dimorphic, with male offspring more strongly affected than females (Alves et al. 2020; Glendining et al. 2018).

EF is strongly mediated by neuronal activity within the prefrontal cortex (PFC), which is heavily innervated by cholinergic fibers that originate predominantly in the basal forebrain (comprising the nucleus basalis, septum, substantia innominata and the diagonal band of Broca) (Bloem et al. 2014). Release of acetylcholine (ACh) in the PFC is associated with performance on attention, working memory, and delayed memory (Bloem et al. 2014; Teles‐Grilo Ruivo et al. 2017). Decreased connectivity and altered neuronal activity have been reported in the PFC of children born to obese or overweight mothers (Li, Andres, et al. 2016; Shapiro et al. 2020; Verdejo‐Roman et al. 2019). On the other hand, increased neurogenesis and neuronal proliferation have also been observed in the cortex of fetal and young adult mice born to HF‐fed mothers (Niculescu and Lupu 2009; Rincel et al. 2018). Thus, similar to the conflicting reports of the effect of maternal obesity on measures of EF, there is similar uncertainty around the impact of perinatal HF feeding on offspring brain structure. In addition, although alterations in dopaminergic, serotonergic, cannabinoid, and mu‐opioid systems have been reported in the PFC of offspring born to HF‐fed mothers (Contu et al. 2022; Lippert et al. 2020; Rincel et al. 2016; Thompson et al. 2017; Vucetic et al. 2010), whether a similar effect is observed in the cholinergic system is not yet known.

The purpose of this study was to investigate the relative contribution of peri‐ and postnatal HF feeding, alone and in combination, on EF and underlying neurobiological changes in the PFC of adult and aged male and female offspring.

## 2 Materials and Methods

### 2.1 Animal Model

Detailed information about the mouse model of pre‐ and post‐weaning HF feeding, including dam and offspring body weight, food consumption, and other metabolic characteristics has been published previously (Contu et al. 2022, 2019). Proven dams were used, in which female C57BL/6J mice were maintained on a standard rodent chow and bred when mice were ~2 months old. Once the first litter was weaned, dams were fed either a control (C) 10% fat or 45% high fat (HF) diet (Special Diet Services, UK) for 4 weeks before mating and during gestation and lactation. Dams fed the C or HF diet who did not get pregnant were excluded from the experiment. C and HF diets were matched for macro and micro minerals, vitamins, and amino acid concentrations (Table S1). Stud C57BL/6J mice were fed the C diet throughout the experiment. At weaning (P28), male and female offspring were randomly assigned to either the C or HF diet, generating four experimental groups: C/C, C/HF, HF/C, HF/HF, representing the pre‐ and post‐weaning diet, respectively. Offspring were fed *ad libitum* until the start of the behavioral experiments and kept on the post‐weaning diet until sacrifice. At the start of both testing periods, both C/HF and HF/HF mice weighed significantly more than C/C and HF/C animals (*p* < 0.001, Tables S2 and S3) (Contu et al. 2022). All experiments were approved by the Open University Animal Welfare and Ethics Review Board and the UK Home Office (PPL 70/8507).

### 2.2 Behavioral Training Protocol

Mice were maintained on a 12 h light: dark cycle (lights on at 7 am) and testing was carried out during the light period. Learning was assessed in Bussey‐Saksida Mouse Touch Screen Chambers (Campden Instruments, Loughborough, UK) using Strawberry milkshake (Yazoo, FrieslandCampina UK, Horsham, UK) as the operant reinforcer. Mice were food restricted to ~90% to 85% of their free‐feeding weight for the duration of testing. After testing was complete, animals were again allowed *ad libitum* access to their diet. Details of the schedule of restriction and *ad libitum* feeding have been published previously (Contu et al. 2022). All animals began testing at 6 months of age and underwent 2 days of habituation in the chambers, followed by an initial touch protocol (1 day, 15 trials over 60 min). Animals in group A did not undergo additional behavioral testing and were rested until sacrifice at 10 months of age. Animals in group B underwent a longitudinal testing protocol starting with the fixed‐ratio (FR) task, followed by progressive ratio (PR) and the two‐choice paired visual discrimination reversal (PVDR) task. After completion of the PVDR task, group B animals were rested until 12 months of age and then re‐tested on the PR and PVDR tasks. Group B mice were then rested until sacrifice at 16 months old. Data from the PR task have been published previously (Contu et al. 2022).

### 2.3 Two‐Choice Paired Visual Discrimination Reversal Task (PVDR) Task

The PVDR task was used to assess offspring executive function, including memory processing and set shifting (Dickson et al. 2014). Mice were simultaneously presented with two shapes (at 6 months = marble + fan; at 12 months = flower + flash), pseudo‐randomly located on the left or right side of the touchscreen. During the acquisition phase, animals had to identify the rewarded stimulus (S+) to receive the milkshake. Touching the unrewarded stimulus (S−) resulted in an inter‐trial timeout and the spatial location of the stimuli was held constant in subsequent trials until the mice made a correct choice. Mice were given 15 cumulative days (30 trials over 60 min/day) to reach criteria (≥ 80% correct stimulus over 2 consecutive days). Animals that reached criteria before 15 days were rested and underwent weekly “refresh” sessions, while those that did not reach criteria were assigned a ‘days to criteria’ number of 16 days. Mice that achieved criteria underwent a reversal phase, where the S+ and S− stimuli were swapped, and animals were given up to 10 days to achieve criteria for the newly rewarding stimulus. Mice were omitted from reversal testing if they did not reach criteria during the acquisition phase or failed to achieve ≥ 80% accuracy during refresh sessions. The total number of mice tested (*n* = 19–37/group) and the proportion of mice in each diet group who reached criteria during acquisition and reversal are shown in Tables S2 and S3.

### 2.4 Tissue Collection

Tissue collection took place during the light period. All mice were deeply anesthetized with an overdose of sodium pentobarbital and then perfused intracardially with 0.01 M phosphate buffered saline (PBS). For frozen tissues, brains underwent rapid gross dissection to isolate the PFC (containing all layers of the medial and lateral orbital area, prelimibic region and infralimbic region), which was snap frozen on dry ice and kept at −80°C until use. For fixed tissues, mice were additionally perfused with 4% paraformaldehyde, and brains were cryopreserved in 30% sucrose and then sectioned on a cryostat (20 μm thickness), with free‐floating sections stored at −20°C in anti‐freeze solution.

### 2.5 RT‐qPCR

Frozen tissues (*n* = 5/group) were placed in RNAlater‐ICE (Fisher Scientific, Loughborough, UK) and transferred to −20°C for at least 2 days before being processed for RNA extraction using the RNeasy Plus Universal Mini Kit (Qiagen, Manchester, UK). cDNA was synthesized using the Applied Biosystems High‐Capacity cDNA Reverse Transcription Kit (Fisher Scientific). KiCqStart SYBR Green Primers (Merck, Gillingham, UK) were used to quantify mRNA levels of serotonin 5‐HT1A (*Htr1a*) and 5‐HT2A (*Htr2a*) receptors, glutamate decarboxylase 1 (*Gad1*) and 2 (*Gad2*), solute carrier family member 17 (*Slc17a7*) and choline acetyltransferase (*Chat*). Expression of the gene of interest was normalized to β‐actin. Relative gene expression was quantified using the 2^−∆∆*Ct*^ method. Comparison between diet groups used the C/C group as the reference group. For age‐related comparisons, values from the 10‐month‐old mice were used as the reference group.

### 2.6 Western Blotting

Frozen tissues (*n* = 5/group) were homogenized in Ripa lysis buffer [150 mM NaCl, 0.1% SDS, 50 mM NaF, 1 mM NaVO3, 20 mM Tris–HCl (pH 8.0), 1 mM EDTA, 1% Igepal] containing a protease inhibitor cocktail (Merck Millipore, Watford, UK). Homogenates were centrifuged (10,000 *g*, 10 min, 4°C) and supernatants were frozen at −80°C until further use. Proteins (30–40 μg) were separated on 4%–20% Tris‐glycine gels (Fisher Scientific) and transferred onto a nitrocellulose membrane. Membranes were blocked in 8% non‐fat milk and incubated overnight at 4°C with antibodies against neuronal nuclear protein (NeuN, 1:500, Cell Signaling Technology (CST), Leiden, The Netherlands), synaptophysin (1:1000, CST), post‐synaptic density 95 (PSD95, 1:350, CST) and ChAT (1:500, Merck, Feltham, UK). Protein loading was confirmed by stripping and re‐probing membranes with anti‐glyceraldehyde‐3‐phosphate dehydrogenase (GAPDH, 1:50,000, Merck). Data were generated from replicates of two blots. Densitometry of immunoblots was quantified using Fiji software (NIH, Maryland, USA), with the optical density ratio of protein levels normalized to GAPDH levels and presented as a percentage of the expression in C/C mice.

### 2.7 Immunohistochemistry

Tissue sections (*n* = 5/group) were washed in 0.01 M PBS, treated with boiling 10 mM sodium citrate (containing 0.05% triton ×100) and incubated with 15% goat serum before being incubated overnight at 4°C with anti‐ChAT (1:50, CST). The following day, sections were washed in PBS and incubated with anti‐goat AlexaFluor488 (1:200, Fisher) for 2 h at room temperature. Subsequently, sections were washed in PBS, incubated with anti‐NeuN (1:1500, CST, overnight at 4°C) and developed using anti‐rabbit AlexaFluor555 (1:200, Fisher). Tissues were then treated with 0.3% Sudan Black (Merck Life Science) for 10 min at RT and coverslipped with Mowiol mounting media (Merck Life Science). Microscopy images were captured on the Leica Stellaris confocal microscope using consistent laser power, gain, and intensity (Leica Microsystems GmbH, Germany) and exported to Adobe Photoshop 2025. Images were processed in Fiji to calculate the percentage area covered by ChAT and NeuN staining in the basal forebrain.

### 2.8 Statistical Analysis

Analyses were carried out using GraphPad Prism 10 (San Diego, USA) or R (version 4.3.0). Data were checked for normality using the Shapiro–Wilk test. Outliers were identified and excluded using the ROUT test. Evaluations of sex × diet were carried out using a two‐way ANOVA with a Sidak post hoc test. For age × diet analyses, comparisons were analyzed using the mixed model for repeated measures with a Sidak post hoc test. Kaplan–Meier curves comparing the rate of learning were analyzed using the chi‐square test, with the *p* value adjusted to control for multiple comparisons (*p* < 0.013 considered significant). Correlations between an animal's breakpoint in the PR task (continuous variable) and whether it reached criteria (yes/no) in the acquisition phase of the PVDR task were assessed using a linear mixed effects logistic regression model, fitted to the data using the lme4 package. This model included a random intercept at the individual mouse level, accounting for repeated measures at two time points for each mouse. Independent variables included diet (categorical variable with four factor levels), sex, and motivation. Motivation was quantified as a binary variable, with “low motivation” corresponding to breakpoint ≤ 8.5 and “high motivation” corresponding to breakpoint > 8.5 that were obtained from the PR task. For all analyses, comparisons between C/C and HF/HF mice and between C/HF and HF/C animals were not carried out, as they did not allow for the relative contribution of peri‐ and postnatal HF diet on outcomes to be measured. Data represent mean ± SEM and *p* < 0.05 was considered to be statistically significant, unless otherwise specified.

## 3 Results

### 3.1 Postnatal HF Feeding Impairs Learning of the PVDR Task in Young Adult Offspring

**FIGURE 1:**
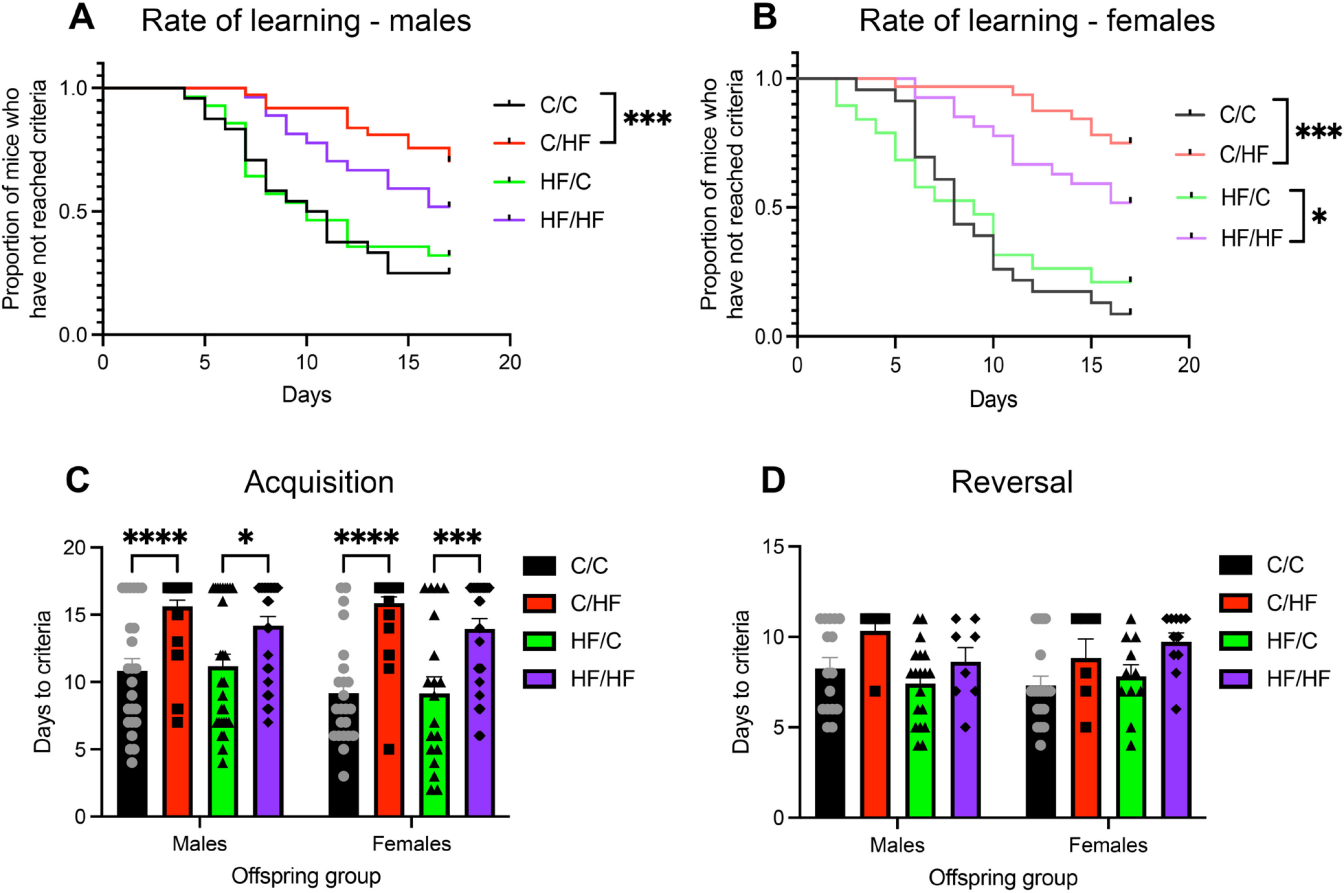
(A, B) Kaplan–Meier curves showing the proportion of 10‐month‐old male (A) and female (B) mice in each diet group who reached criteria during the acquisition phase of the PVDR task as a function of time. *p < 0.05, ***p < 0.001, Chi‐square test adjusted for multiple comparisons. (C, D) The number of days taken by male and female offspring to reach criteria during the acquisition (C) and reversal (D) phase of the PVDR. *p < 0.05, ***p < 0.001, ****p < 0.0001, two‐way ANOVA with Sidak's post hoc.

To determine the effect of peri‐ and postnatal HF on a measure of EF, male and female C/C, C/HF, HF/C, and HF/HF offspring were tested in the PVDR task at 6 and 12 months of age. During the acquisition phase, 6‐month‐old C/HF males learned at a slower rate (Figure 1A) and took significantly longer to reach criteria than male C/C mice (Figure 1C). In addition, only 24% of the male C/HF cohort reached criteria, compared to 83% of C/C mice (*p* < 0.0001, Table S2). No significant differences in days to criteria or the rate of learning were observed between male C/C and HF/C mice or between C/HF and HF/HF males (Figure 1A,C). Similarly, although the rate of learning of male HF/C offspring appeared to be faster than HF/HF mice (unadjusted *p* = 0.04), this difference was not maintained when the *p* value was adjusted for multiple comparisons (Figure 1A). Of the male mice that progressed to the reversal phase of testing (Table S2), no differences were noted between diet groups in days to criteria during reversal (Figure 1D).

In female offspring, both C/HF and HF/HF mice learned the task more slowly than C‐fed mice (Figure 1B). Days to criteria during the acquisition phase were also significantly higher in both C/HF and HF/HF mice compared to C/C and HF/C animals, respectively (Figure 1C). Similarly to male mice, significantly fewer total C/HF females (18%) reached criteria compared to C/C females (91%) (*p* < 0.013, Table S3). No differences were noted in the rate of learning or days to criteria between females fed the same postnatal diet (Figure 1B,C). Although days to criteria in the reversal data showed a similar pattern to that of the acquisition phase, no significant differences were noted between female diet groups (Figure 1D).

### 3.2 Aged HF/C Males Show Better Performance in PVDR Task Than C/C Males

**FIGURE 2:**
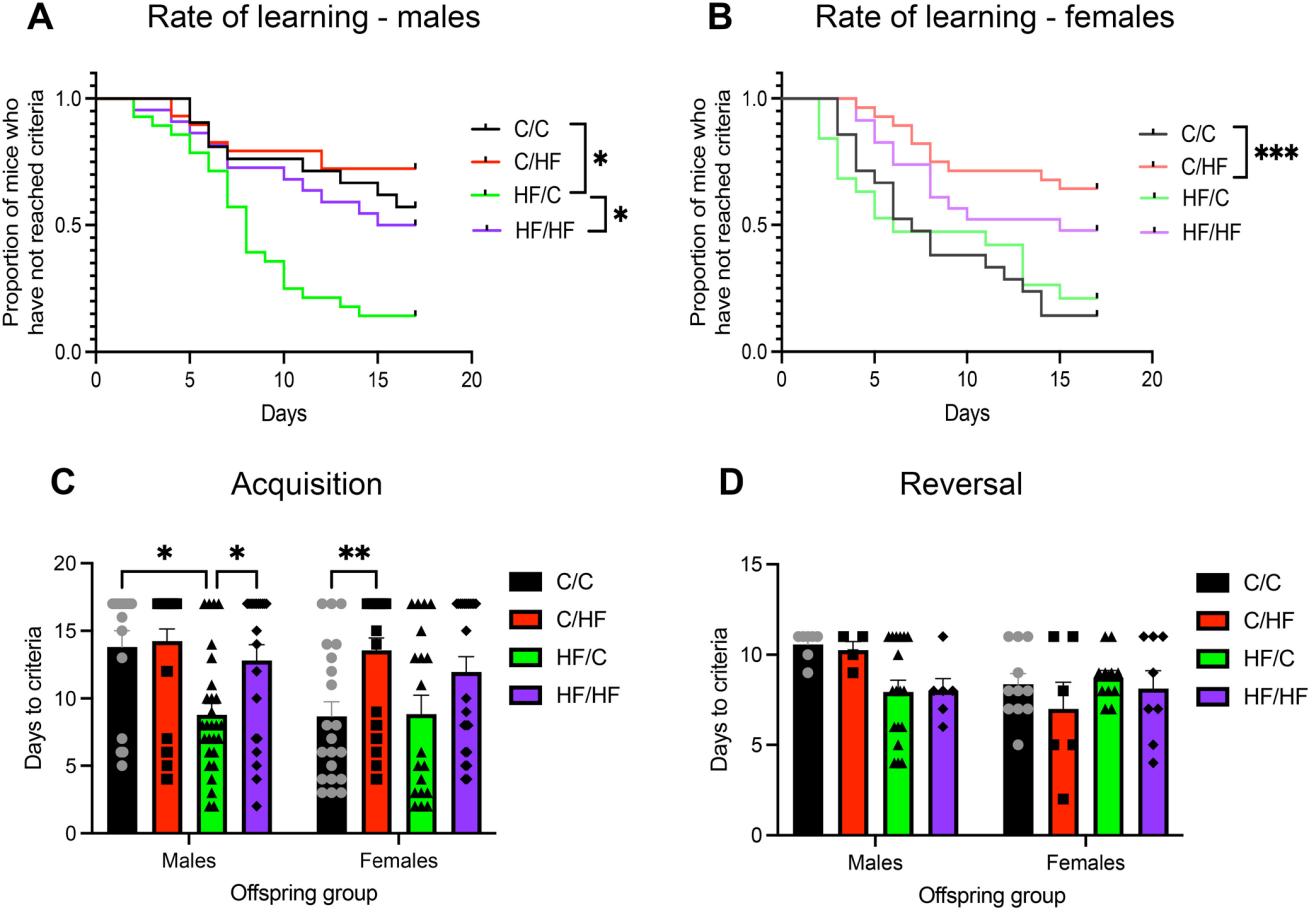
(A, B) Kaplan–Meier curves showing the proportion of 16‐month‐old male (A) and female (B) mice in each diet group who reached criteria during the acquisition phase of the PVDR task as a function of time. *p < 0.05, ***p < 0.001, Chi‐square test adjusted for multiple comparisons. (C, D) The number of days taken by male and female offspring to reach criteria during the acquisition (C) and reversal (D) phase of the PVDR. *p < 0.05, **p < 0.01.

At 12 months of age, the rate of learning and days to criteria were similar between C/C and C/HF mice during acquisition (Figure 2A,C). However, HF/C males learned the task more quickly and were significantly faster at reaching criteria compared to both C/C males and HF/HF mice (Figure 2A,C). Moreover, a significantly larger proportion of HF/C males (86%) attained criteria compared to C/C (43%, *p* < 0.013) and HF/HF males (50%, *p* < 0.013) at this age (Table S2). By contrast, performance was similar between 12‐month‐old C/C and HF/C females, while C/HF females still took significantly longer than C/C mice to learn the task and to reach criteria (Figure 2B,C). Although a limited number of 12‐month‐old mice were progressed onto the reversal portion of the task (Tables S2 and S3), no differences were observed between diet groups in days to criteria (Figure 2D).

Age comparisons found that C/C male mice took approximately 5 days longer to reach criteria at 12 months of age than when they were 6 months old (*p* < 0.05, Figure S1A). Time to criteria did not differ significantly between young and aged mice in the other male diet groups. Performance of the female mice across all diet groups was also unaffected by age (Figure S1B).

### 3.3 Effect of Motivation on Performance in PVDR Task

We have previously observed that motivation for the milkshake reward during the PR task was significantly lower in young, but not aged, C/HF and HF/HF animals (Contu et al. 2022). In the current experiment, latency to collect reward during acquisition was significantly higher in 6‐month‐old male and female C/HF and HF/HF offspring compared to C‐fed animals (Figure S1C). By contrast, HF/HF male mice collected the reward more quickly than C/HF animals (Figure S1C). At 12 months of age, reward collection latency was similar between C/C and C/HF male mice, but HF/C males had significantly faster collection latencies than both C/C and HF/HF males (Figure S1D). Aged female C/HF animals remained significantly slower to collect reward than C/C and HF/HF females (Figure S1D). Age‐related comparisons found that 12‐month‐old male C/C mice had significantly increased reward collection latency compared to latency at 6 months of age, while aged C/HF had lower latency than when they were younger (Figure S1E). No differences were noted between young versus aged female offspring in any diet groups (Figure S1F).

To determine if motivation for the reward influenced performance on the PVDR task, regression analyses were carried out between the breakpoint in the PR task and whether the same mice reached criteria in the PVDR test. A significant correlation was noted between high motivation and likelihood of achieving criteria (OR: 3.0 (95% CI: 1.7–5.1), *p* < 0.0001). However, after adjustment for breakpoint and offspring sex, postnatal HF diet remained a statistically significant predictor of performance on PVDR (*p* < 0.0001), suggesting that the observed differences in days to criteria between offspring diet groups were not due to differences in motivation for the reward.

### 3.4 Markers of Acetylcholine Are Altered in the PFC and Medial Septum of HF/C Mice

**FIGURE 3:**
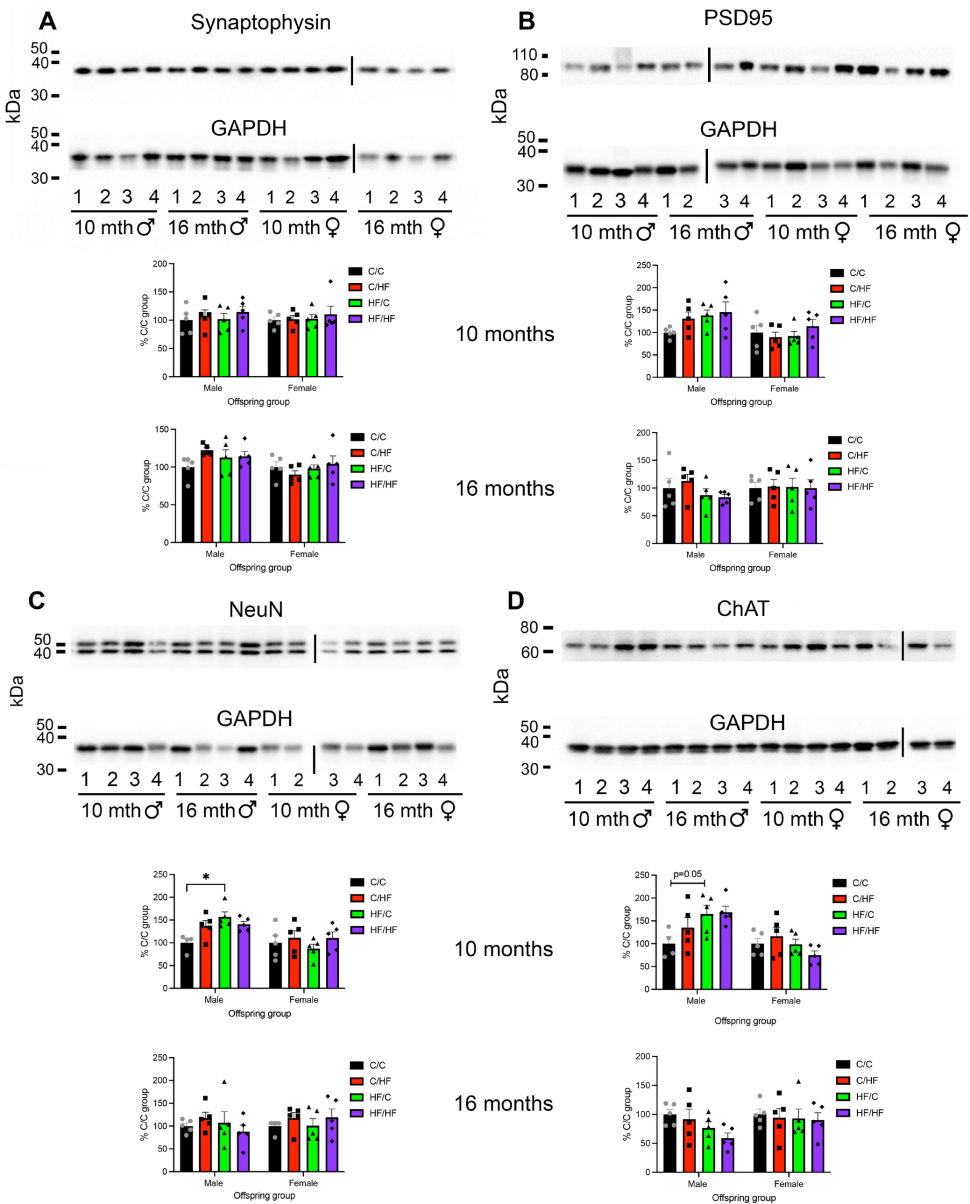
(A–D) Representative Western blots and quantification of protein levels of synaptophysin (A), PSD95 (B), NeuN (C) and ChAT (D) in the prefrontal cortex of 10‐ to 16‐month‐old male and female offspring groups. Numbering under the blots indicates the diet group, 1 = C/C, 2 = C/HF, 3 = HF/C, 4 = HF/HF. Vertical lines indicate where samples were loaded on separate gels. *p < 0.05, two‐way ANOVA with Sidak's post hoc.

To determine if the differences in performance on the PVDR task were due to alterations in neuronal density, PFC samples collected from 10‐ and 16‐month‐old offspring were processed for markers of neurons and synapses. Levels of the pre‐ and post‐synaptic markers synaptophysin and PSD95, respectively, were unchanged between any diet group at 10 months of age (Figure 3A,B). Levels of the marker of mature neurons NeuN were significantly higher in 10‐month‐old male HF/C mice compared to C/C mice (*p* < 0.05, Figure 3C). No differences in NeuN expression were observed between other male diet groups or between female diet groups. In tissues collected from 16‐month‐old mice, the expression of NeuN, synaptophysin, or PSD95 was unchanged between diet groups (Figure 3A–C). Levels of NeuN, synaptophysin, and PSD95 were also similar between young and aged mice in the same diet groups (Figure S2A–F).

We next determined if the observed increase in neuronal expression in the PFC of HF/C males was associated with selective changes in markers of serotonin, GABA, glutamate, or acetylcholine using RT‐qPCR and Western blotting. Markers of dopamine were excluded from the current analysis because we have previously reported that dopamine receptor 1 (*Drd1*) mRNA expression was significantly increased in the PFC of female C/HF versus C/C mice, while *Drd2* gene expression and protein levels were significantly higher in the PFC of 10‐month‐old HF/C male mice compared to C/C offspring (Contu et al. 2022).

In 10‐month‐old offspring, mRNA levels of the serotonin 5‐HT1A receptor (*Htr1a*) and the 5‐HT2A receptor (*Htr2a*) were similar between diet groups (Table 1). The expression of GABAergic markers, *Gad1* and *Gad2*, and the glutamate marker VGLUT1 (*Slc17a7*) were also similar between diet groups (Table 1). Analysis of cholinergic markers found that *Chat* mRNA levels were approximately 4‐fold higher in male HF/C mice compared to C/C animals (Table 1). Protein levels of ChAT were also upregulated in HF/C mice, although this difference bordered on significance (*p* = 0.05) (Figure 3D). *Chat* mRNA and protein levels were unchanged between female diet groups (Table 1 and Figure 3D).

**TABLE 1:** Gene expression in 10‐month‐old male and female offspring diet groups relative to the C/C group.

Genes	Males				Females			
	C/C	C/HF	HF/C	HF/HF	C/C	C/HF	HF/C	HF/HF
*Chat*	**1.6 ± 0.7**	2.8 ± 1.0	5.9 ± 1.5*	3.6 ± 1.8	**1.0 ± 0.2**	1.2 ± 0.6	0.8 ± 0.2	1.0 ± 0.4
*Gad1*	**1.0 ± 0.1**	1.9 ± 0.4	1.2 ± 0.2	1.1 ± 0.2	**1.0 ± 0.1**	1.4 ± 0.2	1.1 ± 0.1	1.3 ± 0.2
*Gad2*	**1.4 ± 0.5**	1.9 ± 0.6	1.3 ± 0.4	1.4 ± 0.5	**1.2 ± 0.3**	0.9 ± 0.4	1.1 ± 0.2	1.0 ± 0.1
*Htr1a*	**1.0 ± 0.04**	0.8 ± 0.3	0.8 ± 0.1	1.2 ± 0.2	**1.0 ± 0.1**	0.8 ± 0.2	0.9 ± 0.2	1.0 ± 0.09
*Htr2a*	**1.1 ± 0.3**	0.9 ± 0.3	1.1 ± 0.1	0.9 ± 0.2	**1.0 ± 0.04**	1.0 ± 0.05	1.0 ± 0.04	1.4 ± 0.2
*Slc17a7*	**1.0 ± 0.1**	0.9 ± 0.2	0.8 ± 0.2	1.0 ± 0.1	**1.1 ± 0.2**	0.9 ± 0.1	1.0 ± 0.1	1.2 ± 0.2

* *p* < 0.05 versus C/C, bold indicates the reference group.

In 16‐month‐old mice, mRNA expression of *Htr1a*, *Htr2a*, *Gad1, Gad2*, *Slc17a7*, and *Chat* was similar between diet groups (Table 2). Comparison between adult and aged mice found decreased *Slc17a7* levels in 16‐month‐old male HF/HF offspring, but no significant differences were noted between other genes (Table S4). Although *Chat* gene expression was unaltered by age, ChAT protein levels were significantly higher in 16‐month‐old C/C male mice compared to 10‐month‐old mice, while ChAT expression was stable in females (Figure S2G,H).

**TABLE 2:** Gene expression in 16‐month‐old male and female offspring diet groups relative to the C/C diet group.

Genes	Males				Females			
	C/C	C/HF	HF/C	HF/HF	C/C	C/HF	HF/C	HF/HF
*Chat*	**1.9 ± 1.5**	1.0 ± 0.4	4.9 ± 3.6	1.3 ± 0.5	**1.3 ± 0.5**	6.8 ± 2.5	1.6 ± 0.3	6.8 ± 3.6
*Gad1*	**1.2 ± 0.4**	1.2 ± 0.3	1.8 ± 0.6	1.7 ± 0.8	**1.1 ± 0.3**	1.1 ± 0.1	1.1 ± 0.4	1.6 ± 0.3
*Gad2*	**1.3 ± 0.4**	0.9 ± 0.2	1.9 ± 0.5	1.7 ± 0.8	**1.0 ± 0.1**	1.5 ± 0.2	0.9 ± 0.3	1.3 ± 0.5
*Htr1a*	**1.1 ± 0.2**	1.1 ± 0.1	1.2 ± 0.2	1.2 ± 0.3	**1.0 ± 0.1**	1.2 ± 0.1	1.0 ± 0.1	1.1 ± 0.2
*Htr2a*	**1.0 ± 0.1**	0.8 ± 0.2	1.3 ± 0.1	1.2 ± 0.2	**1.0 ± 0.1**	1.2 ± 0.06	1.1 ± 0.04	1.1 ± 0.09
*Slc17a7*	**1.1 ± 0.2**	0.9 ± 0.1	0.8 ± 0.2	0.6 ± 0.1	**1.0 ± 0.2**	0.7 ± 0.08	0.9 ± 0.2	0.9 ± 0.2

*Note:* Bold indicates the reference group.

**FIGURE 4:**
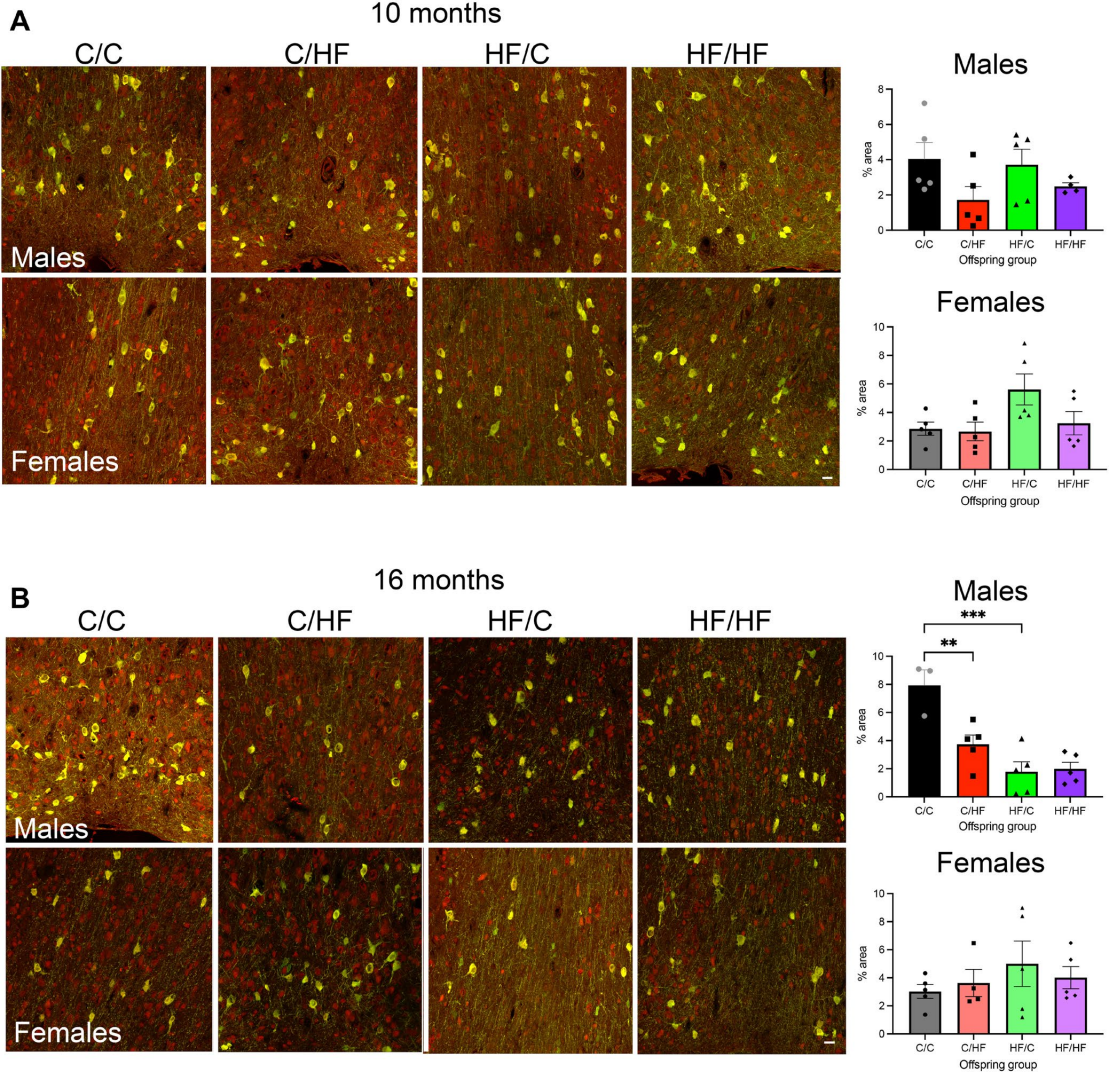
(A, B) Photomicrographs of ChAT (green) and NeuN (red) immunohistochemistry in the medial septum (MS) and diagonal band of Broca (DBB) of 10‐month‐old (A) and 16‐month‐old (B) male and female offspring. Co‐localization of ChAT and NeuN expression is indicated by yellow staining. Graphs showing % area of the MS/DBB covered by ChAT‐positive cells and fibers. Scale bars = 20 μm. **p < 0.01, ***p < 0.001, two‐way ANOVA with Sidak's post hoc.

Given the observation that cholinergic markers in the PFC showed the most prominent differences between diet groups, we examined if this was due to differences in the density of cholinergic cell bodies and fibers originating from the medial septum (MS) and diagonal band of Broca (DBB). ChAT density and NeuN expression were both similar between diet groups in 10‐month‐old male or female mice (Figure 4A and Figure S3A,B). However, in 16‐month‐old mice, cholinergic expression was significantly decreased in male C/HF and HF/C versus C/C mice (Figure 4B). NeuN expression was not significantly different in the same animals, suggesting that the ChAT changes were not due to a general loss of neurons (Figure S3A,C). No differences in ChAT or NeuN expression were noted in the MS/DBB of aged female mice (Figure 4A,B and Figure S3D). Age comparisons showed higher ChAT and NeuN density in 16‐month‐old C/C males versus 10‐month‐old C/C animals (Figure S3E,G), but no other differences were noted between other diet groups. ChAT and NeuN density were also similar between young and old female groups (Figure S3F,H).

## 4 Discussion

Very little is currently known about the long‐term impact of perinatal HF diet, alone or in combination with postnatal HF feeding, on EF across the life course. We found that the performance of adult animals in the PVDR task was strongly influenced by the postnatal diet, with fewer C/HF and HF/HF mice reaching criteria and taking longer to do so compared to C/C and HF/C animals. Although perinatal HF exposure did not influence cognition at 6 months of age, it appeared to protect against the age‐related decline in EF performance that was observed in C/C males. This was underscored by increased cholinergic expression in the PFC of 10‐month‐old male HF/C mice. These results suggest that prolonged consumption of a HF diet negatively impacts EF, but exposure to the same diet during gestation and/or early life alone may be beneficial in older age, possibly due in part to increased cholinergic innervation of the PFC.

Earlier work has suggested that obesity‐associated decreases in appetitive motivation may impair learning in tasks with hedonic food rewards (Harb and Almeida 2014). We have previously observed that motivation for the milkshake in the PR task was significantly lower in young C/HF and HF/HF mice compared to C‐fed mice, but that this effect was lost as the animals aged (Contu et al. 2022). In the PVDR task, latency to collect reward was higher in 6‐month‐old C/HF and HF/HF mice, suggesting decreased motivation similar to that seen in the PR task. However, we did not observe a clear association between motivation and EF. For example, days to criteria were similar between young male C/HF and HF/HF mice, despite a faster reward collection in HF/HF mice. In addition, the rate of learning did not differ between young versus old C/HF males, even as collection latency was significantly lower in 12‐month‐old mice. Finally, the effect of diet on days to criteria remained significant after statistical adjustment for level of motivation. Thus, although we cannot dismiss a potential contribution of decreased motivation to poorer EF in HF‐fed mice, it did not mask the independent effect of the diet.

The poor performance of 6‐month‐old C/HF and HF/HF mice on the PVDR task is consistent with previous findings in humans and animals. Consumption of diets high in sFA is associated with reduced EF and increased risk of cognitive impairment in both young and middle‐aged adults (Francis and Stevenson 2013; Kalmijn et al. 2004). In animal studies, Evans et al. reported poorer spatial learning and decreased cued memory recall in young adult mice fed a 60% fat diet compared to those fed an 18% fat diet (Evans et al. 2024). Intake of sFAs is also associated with a greater decline in memory, attention, and processing speed during normal aging (Francis and Stevenson 2013; Okereke et al. 2012). In the current study, the performance of HF‐fed mice in the PVDR task was unvaried between 6‐ and 12‐months of age. It is possible that this is due to a floor effect, whereby the HF‐fed mice were unable to learn the PVDR task at either age. This also meant that very few C/HF and HF/HF mice were progressed to the reversal phase of the task, and this limited our ability to assess cognitive flexibility in these mice. Of the HF‐fed animals that were tested in reversal, their performance was similar to C‐fed mice, suggesting that EF was intact in those animals. However, alternate tests are needed to determine the impact of post‐natal HF diet on reversal learning. Interestingly, work by Kesby et al. (2015) found that spatial memory performance of 12‐month‐old mice was similar between mice fed a control and HF diet, and they concluded that adult mice were more susceptible than aged mice to the physiological effects of HF diet consumption. This further highlights the important influence of HF exposure during key early neurodevelopmental periods on long‐term cognitive function. In support of this, we also observed that approximately twice as many mice in the HF/HF group (~50%) reached criteria during acquisition compared to C/HF animals (~25%), regardless of age. This supports the “mis‐match” DOHaD hypothesis that perinatal adaptations made in response to an obesogenic environment may be detrimental when the in utero and postnatal environments differ, but advantageous when the offspring are reared in similar postnatal conditions (Calkins and Devaskar 2011).

The effect of maternal obesity on measures of EF in their offspring is still debated. Clinical and epidemiological studies have reported poorer problem‐solving and inhibitory control, as well as lower intelligence quotient in 4 to 6‐year‐old children born to overweight or obese mothers (Menting et al. 2018; Oken et al. 2021; Pugh et al. 2015). Lower cognitive performance is also observed in children as a function of maternal weight gain during pregnancy (Huang et al. 2014; Li, Fallin, et al. 2016; Tavris and Read 1982), although the opposite has also been reported (Gage et al. 2013). Young adult rodent offspring (11–16 weeks old) born to dams fed a HF diet during gestation and lactation perform worse on attentional threshold and reversal tasks, including increased impulsivity, compared to offspring of lean mothers (McKee et al. 2017; Smith et al. 2020). Spatial memory performance is also decreased in offspring of HF‐fed dams (Cordner et al. 2019; Graf et al. 2016) and this can persist across multiple generations (Lin et al. 2021). However, other studies have reported a positive effect of early HF exposure on various aspects of learning and memory. For example, better spatial memory performance has been reported in young male adult (~3 months old) rats and *Peromyscus* mice born to dams fed a 20% to 60% fat diet during gestation and lactation (Bilbo and Tsang 2010; Johnson et al. 2017). Similarly positive effects on memory have also been noted in 11 to 15 weeks old piglets exposed to a HF diet either during the prenatal or pre‐ and postnatal periods (Clouard et al. 2016; Val‐Laillet et al. 2017). More recently, Di Meco and Pratico found that 18‐month‐old mouse offspring born to mothers fed a 42% fat diet during gestation had better spatial memory recall compared to offspring of dams fed a 13% fat diet (Di Meco and Pratico 2019).

In the present study, days to criteria and rate of learning were similar between 6‐month‐old HF/C and C/C mice, suggesting that perinatal exposure did not significantly influence EF in young adulthood, as measured by the PVDR task. However, whilst C/C males, but not females, showed an age‐related decline in performance, the days to criteria during task acquisition were consistent between 6 and 12 months of age in HF/C males. This is consistent with previous findings that male offspring neurobiology and/or behaviors are more sensitive than females to perinatal HF diet exposure (Alves et al. 2020; Bordeleau et al. 2021, 2020; Gemici et al. 2021; Lippert et al. 2020). This also suggests that early life exposure to a 45% fat diet may be beneficial in preserving EF in old age, relative to mice fed a 10% fat diet. The discrepancies between our results and those that have reported a negative impact of maternal and/or perinatal HF exposure may relate to differences in diet composition (e.g., ratio of sFA to unsaturated FAs), timing of maternal feeding (e.g., prior to mating, during gestation or lactation only), tests used to assess EF, as well as the sex and age of offspring at testing (Contu and Hawkes 2017). Additional work is needed to disentangle the contribution of each of these features on offspring outcomes and their relevance to human health. Relatedly, our results also highlight the need for additional longitudinal studies to determine the effect of maternal obesity or perinatal HF feeding on cognitive function into old age.

The PFC plays a key role in the regulation of EF and is heavily innervated by cholinergic fibers that originate predominantly in the basal forebrain, although some midbrain nuclei and cortical interneurons also provide cholinergic innervation (Bloem et al. 2014). Release of ACh in the PFC contributes to attentional control and goal‐directed behaviors, and damage to the medial and dorsolateral PFC is associated with dysfunctions in planning, inhibition, and flexible learning (Takeuchi et al. 2013). Additionally, synaptic density and spine volume in the dorsolateral PFC are predictive of task learning performance (Dumitriu et al. 2010). A recent imaging study also found that the free‐water volume fraction of the basal forebrain and ACh concentrations in the PFC were associated with performance on attention, working memory, and delayed memory, and EF in Parkinson's disease (Crowley et al. 2024).

Human MRI studies have reported decreased cross‐hemispheric connectivity of the PFC in the fetal brains of mothers with elevated BMI (Norr et al. 2021). Altered neuronal resting state activity and lower mean diffusivity have also been observed in the PFC of children of obese or overweight mothers (Li, Andres, et al. 2016; Verdejo‐Roman et al. 2019). In animal studies, decreased expression of genes that regulate neurogenesis and synaptic plasticity, such as *Gadd45b*, *Mecp2*, and *Bdnf*, has been found in the PFC of fetal mice whose mothers were fed a 45% diet before mating and during gestation (Glendining et al. 2018; Urbonaite et al. 2022). Conversely, other research has reported that in utero exposure to a HF diet increased the proliferation of neural progenitors in the cortex of fetal mice (Niculescu and Lupu 2009) and protected against perinatal stress‐induced loss of spine density and dendritic atrophy in adolescent and adult rats (Rincel et al. 2018). Maternal HF feeding has also been associated with increased expression of PSD95 in the cortex of 18‐month‐old mice (Di Meco and Pratico 2019).

We observed that 10‐month‐old male HF/C mice had more overall neurons in the PFC compared to C/C mice, which was underscored by a selective increase in the expression of cholinergic markers. Interestingly, this was not mirrored by increased cholinergic cell density in the MS/DBB, suggesting that either increased arborization or additional innervation arising outside the basal forebrain may have contributed to the observed increase in cortical ChAT expression. Although more work is needed to determine how perinatal HF exposure influences neurogenesis, it is notable that, in addition to containing higher concentrations of sFAs (predominantly palmic acid and steric acid), the HF diet used in this study also comprised greater amounts of the monounsaturated FA (MUFA) oleic acid and the polyunsaturated FA (PUFA) linoleic acid, relative to the C diet. Both MUFAs and PUFAs are critical for brain development, and early life deficiencies in n‐3 and n‐6 PUFAs can delay neuronal cell migration (Martinat et al. 2021; Yavin et al. 2009) and impair cognitive flexibility (Yamamoto et al. 1988). In particular, oleic acid, which can also be produced from the desaturation of stearic acid (Bruce and Salter 1996), plays a key role in neural stem cell survival (Kandel et al. 2022) and consumption of oleic acid may provide beneficial cognitive effects in elderly individuals (Sakurai et al. 2021). Thus, exposure to the HF diet during the perinatal period may have provided higher concentrations of key FAs needed for optimal neuronal growth and survival. This may have in turn contributed to sustained performance in the PVDR task across the lifespan of HF/C mice. However, because of the differential testing protocol between mice killed at 10 months versus 16 months of age, we cannot dismiss the possibility that the additional testing may have promoted neuronal or dendritic growth in the aged mice. This may also account in part for the observed increase in ChAT and NeuN expression in the MS/DBB in 16‐month‐old C/C males versus 10‐month‐old C/C animals. Further studies are needed to determine the effect of perinatal HF diet, alone and in combination with postnatal HF feeding, on neuronal development and maturation in behaviorally naïve, aged offspring.

In summary, our results suggest that chronic postnatal HF feeding resulted in impaired EF in young mice, irrespective of the perinatal diet. However, aged male offspring born to mothers fed the HF diet, who are themselves lean, performed better in the PVDR task than mice born to C‐fed mothers, possibly due to increased expression of cholinergic innervation of the PFC. Given the high rates of maternal obesity, these data may have implications for advice given to expectant mothers and children born to obese mothers about the composition and timing of consumption of dietary fats.

## Author Contributions

Experimental design, data collection, and analyses were carried out by L.C. and A.J. Data analyses and manuscript writing was carried out by C.J.H. and C.A.H. All authors contributed to manuscript editing.

## Conflicts of Interest

The authors declare no conflicts of interest.

## Supporting information


**Figure S1:** (A, B) Comparison of days to criteria during the acquisition phase of the PVDR between 6‐ and 12‐month‐old male (A) and female (B) mice. (C–F) Latency to collect the reward during the acquisition phase of the PVDR in male and female offspring tested at 6‐months (C) and 12‐months (D) of age. Age‐related changes in reward collection latency of male (E) and female (F) offspring. *p < 0.05, **p < 0.01, ***p < 0.001, ****p < 0.0001, two‐way ANOVA with Sidak's post hoc.

**Figure S2:** (A–H) Quantification of protein levels of synaptophysin (A, B), PSD95 (C, D), NeuN (E, F) and ChAT (G, H) in the PFC of 10‐ and 16‐month‐old male and female offspring. **p < 0.05, two‐way ANOVA with Sidak's post hoc.

**Figure S3:** (A–D) Quantification of percentage area of the medial septum (MS) and diagonal band of Broca (DBB) that is positive for NeuN staining in 10‐month‐old (A, B) and 16‐month‐old (C, D) male and female offspring. (E–H) Age‐related changes in percent coverage of ChAT‐positive cells and fibers (E, F) and NeuN‐positive cells (G, H) in the MS/DBB of 10‐ and 16‐month‐old male and female offspring. *p < 0.05, two‐way ANOVA with Sidak's post hoc.

**Tables S1–S4:** acel70223‐sup‐0004‐Tables.docx.

## Data Availability

The data that support the findings of this study are available on request from the corresponding author. The data are not publicly available due to privacy or ethical restrictions.
