# Supplementary figures and images for: Peri‐ and Postnatal High Fat Feeding Have Differential Effects on Executive Function and Associated Neurobiology in Aged Male and Female Mice
Source: Aging Cell. 2025 Sep 7;24(11):e70223. doi: 10.1111/acel.70223 (PMC12611268; doi:10.1111/acel.70223)

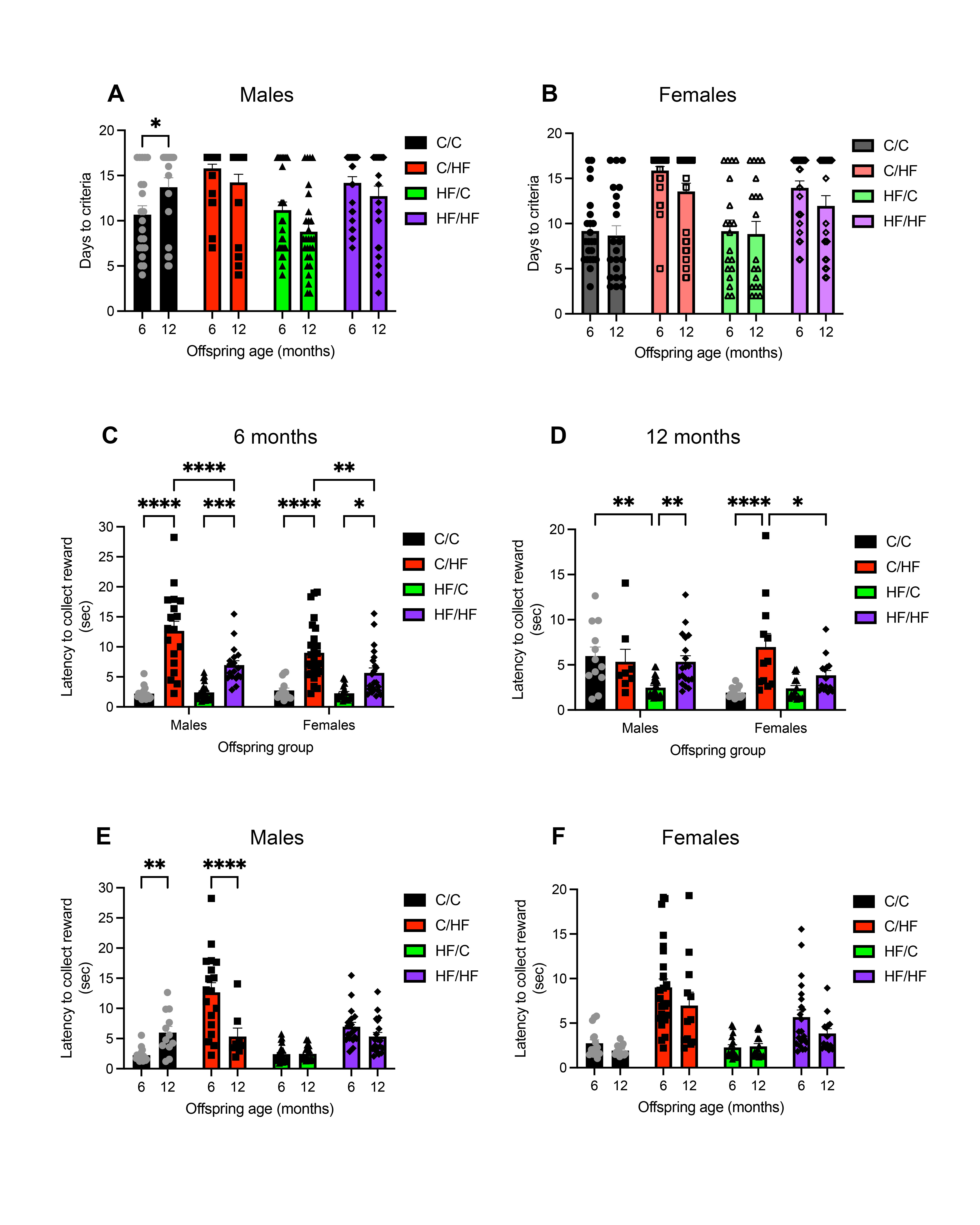

Supplement: Supplementary file 1 — Figure S1: (A, B) Comparison of days to criteria during the acquisition phase of the PVDR between 6‐ and 12‐month‐old male (A) and female (B) mice. (C–F) Latency to collect the reward during the acquisition phase of the PVDR in male and female offspring tested at 6‐months (C) and 12‐months (D) of age. Age‐related changes in reward collection latency of male (E) and female (F) offspring. *p < 0.05, **p < 0.01, ***p < 0.001, ****p < 0.0001, two‐way ANOVA with Sidak's post hoc. [file ACEL-24-e70223-s002.jpg]

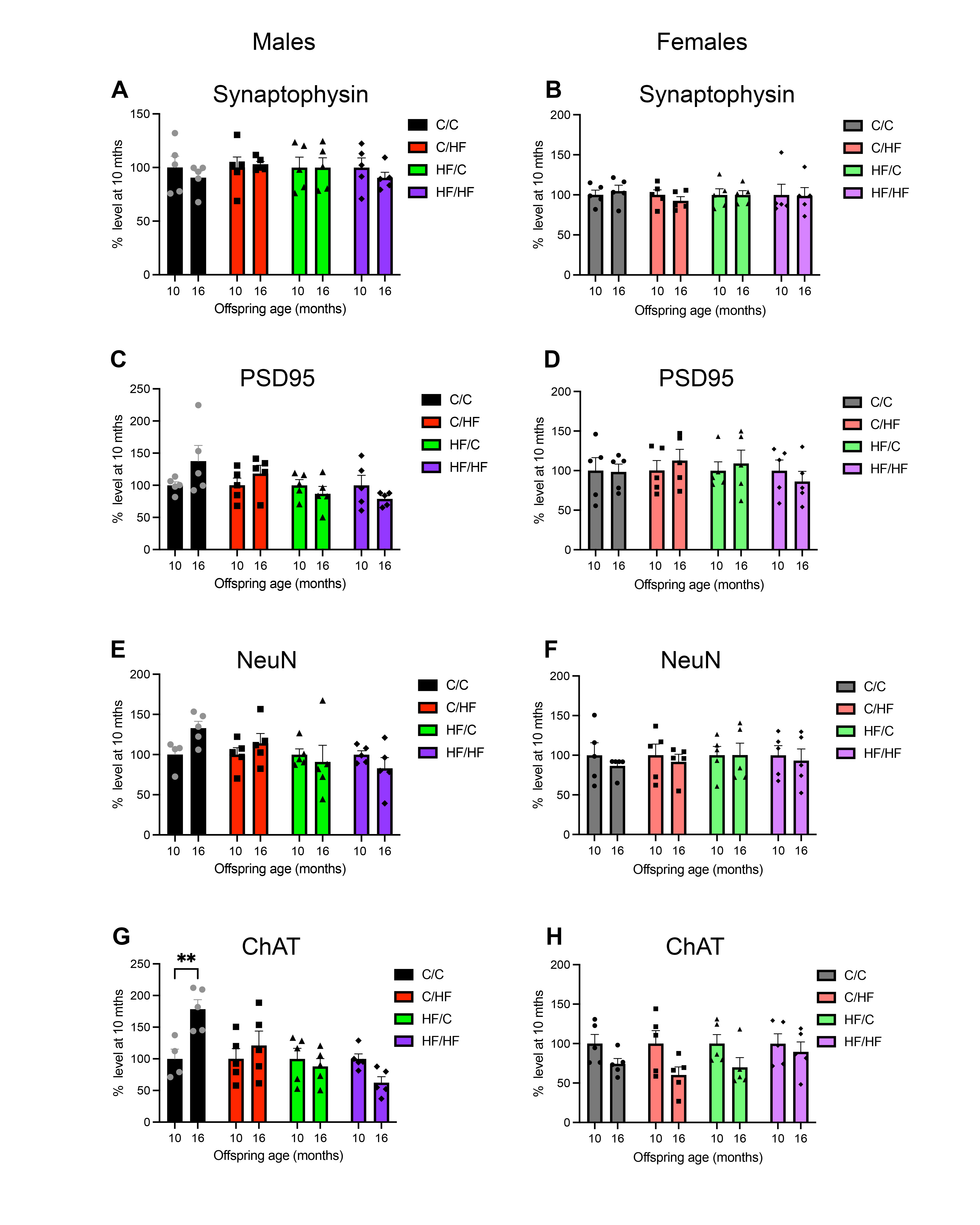

Supplement: Supplementary file 2 — Figure S2: (A–H) Quantification of protein levels of synaptophysin (A, B), PSD95 (C, D), NeuN (E, F) and ChAT (G, H) in the PFC of 10‐ and 16‐month‐old male and female offspring. **p < 0.05, two‐way ANOVA with Sidak's post hoc. [file ACEL-24-e70223-s003.jpg]

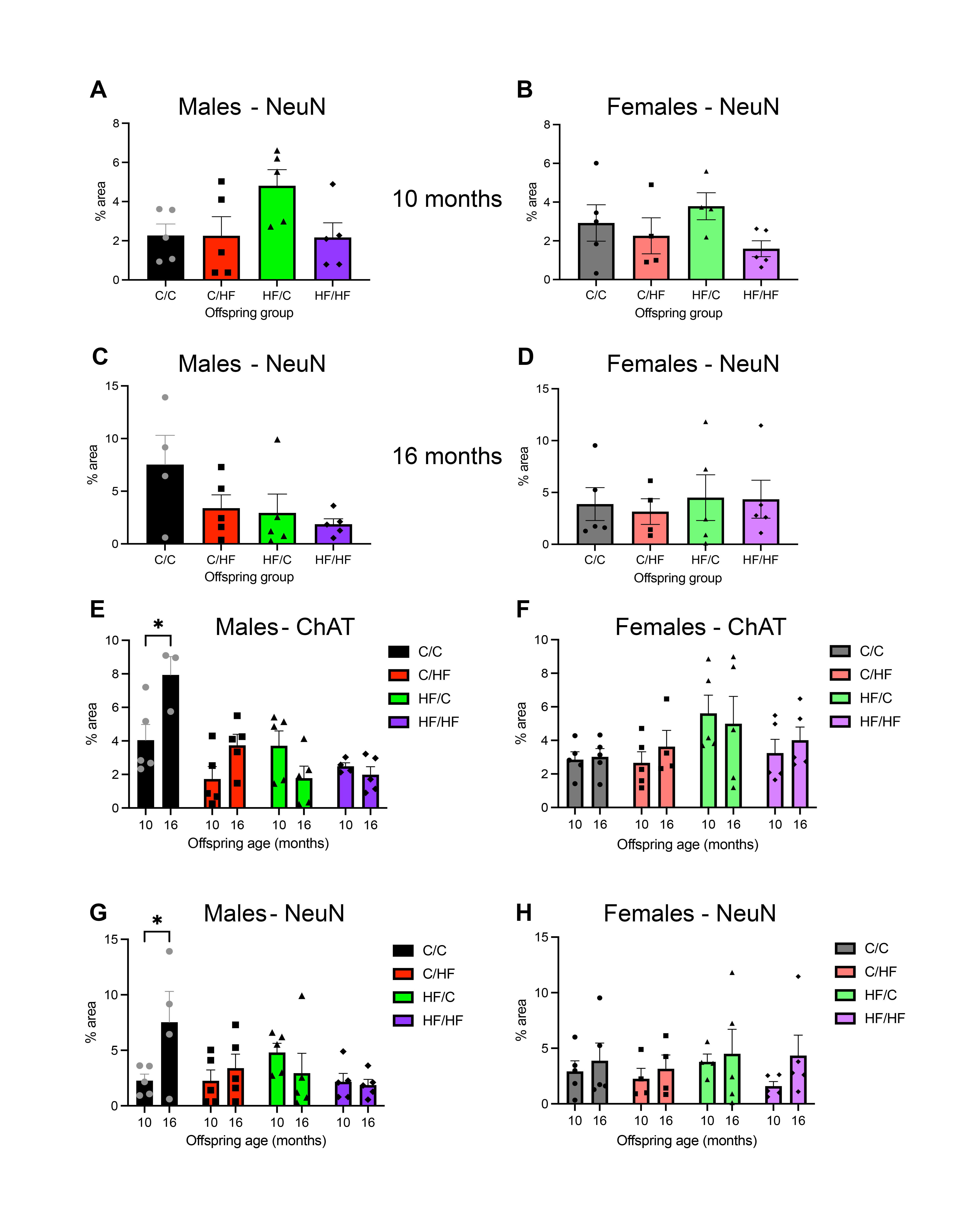

Supplement: Supplementary file 3 — Figure S3: (A–D) Quantification of percentage area of the medial septum (MS) and diagonal band of Broca (DBB) that is positive for NeuN staining in 10‐month‐old (A, B) and 16‐month‐old (C, D) male and female offspring. (E–H) Age‐related changes in percent coverage of ChAT‐positive cells and fibers (E, F) and NeuN‐positive cells (G, H) in the MS/DBB of 10‐ and 16‐month‐old male and female offspring. *p < 0.05, two‐way ANOVA with Sidak's post hoc. [file ACEL-24-e70223-s001.jpg]
