# Supplementary material for: Peri‐ and Postnatal High Fat Feeding Have Differential Effects on Executive Function and Associated Neurobiology in Aged Male and Female Mice
Source: Aging Cell. 2025 Sep 7;24(11):e70223. doi: 10.1111/acel.70223 (PMC12611268; doi:10.1111/acel.70223)
Supplement: Supplementary file 4 — Tables S1–S4: acel70223‐sup‐0004‐Tables.docx. [file ACEL-24-e70223-s004.docx]

**Supplemental Tables**

**Supplemental Table 1** Composition of ingredients in the control and high fat diets

|  | Control diet | High fat diet |
| --- | --- | --- |
| **Ingredients** (% inclusion) |  |  |
| Casein | 21.5 | 26.5 |
| Choline bitartrate | 0.24 | 0.296 |
| L-Cystine | 0.32 | 0.398 |
| Lard | 0 | 18 |
| Rice Starch | 32.44 | 18.43 |
| Cellulose | 5 | 6.164 |
| Soya oil | 3.5 | 4.315 |
| Sucrose | 32.5 | 20.34 |
| Mineral mix | 3.5 | 4.315 |
| Vitamin mix | 1 | 1.233 |
|  |  |  |
| **Proximates** (%) |  |  |
| Crude oil | 3.73 | 21.22 |
| Crude protein | 17.59 | 21.55 |
| Crude fibre | 3.59 | 4.22 |
| Ash | 3.38 | 4.12 |
| Nitrogen free extract | 60.43 | 37.90 |
|  |  |  |
| **Fatty acids** (%) |  |  |
| C12:0 (lauric acid) | 0.07 | 0.03 |
| C14:0 (myristic acid) | 0.12 | 0.29 |
| C16:0 (palmitic acid) | 0.22 | 4.04 |
| C18:0 (steric acid) | 0.13 | 1.8 |
| C14:1 (myristoleic acid) | 0.01 | 0.02 |
| C16:1 (palmitoleic acid) | 0.07 | 0.03 |
| C18:1 (oleic acid) | 0.66 | 6.06 |
| C18:2 (linoleic acid) | 1.26 | 3.53 |
| C18:3 (linolenic acid) | 0.22 | 0.37 |
| C20:4 (arichidonic acid) | 0.01 | 0.01 |
| C22:5 (clupanodonic acid) | 0 | 0 |
|  |  |  |
| **Amino acids** (%) |  |  |
| Arginine | 0.55 | 0.69 |
| Lysine (6) | 1.11 | 1.37 |
| Methionine | 0.43 | 0.53 |
| Cysteine | 0.35 | 0.42 |
| Tryptophan | 0.16 | 0.19 |
| Histidine | 0.4 | 0.49 |
| Threonine | 0.61 | 0.75 |
| Isoleucine | 0.88 | 1.09 |
| Leucine | 1.33 | 1.64 |
| Phenylalanine | 0.73 | 0.9 |
| Valine | 1.06 | 1.3 |
| Tyrosine | 0.73 | 0.9 |
| Taurine | 0 | 0 |
| Glycine | 0.7 | 0.85 |
| Aspartic acid | 0.99 | 1.22 |
| Glutamic acid | 2.87 | 3.53 |
| Proline | 1.2 | 1.47 |
| Serine | 0.66 | 0.82 |
| Hydroxyproline |  | 0 |
| Hydroxylsine |  | 0 |
| Alanine | 0.56 | 0.69 |
|  |  |  |
| **Macro minerals** |  |  |
| Calcium (%) | 0.46 | 0.59 |
| Total Phosphorus (%) | 0.18 | 0.35 |
| Phytate Phosphorus (%) |  | 0 |
| Available Phosphorus (%) |  | 0.35 |
| Sodium (%) | 0.12 | 0.15 |
| Chloride (%) | 0.22 | 0.26 |
| Potassium (%) | 0.46 | 0.42 |
| Magnesium (%) | 0.06 | 0.08 |
|  |  |  |
| **Micro minerals** |  |  |
| Iron (mg/kg) | 44.99 | 55.7 |
| Copper (mg/kg) | 6.67 | 8.22 |
| Manganese (mg/kg) | 10.13 | 12.22 |
| Zinc (mg/kg) | 54.63 | 64.67 |
| Cobalt (μg/kg) | 0 | 0 |
| Iodine (μg/kg) | 194.04 | 238.95 |
| Selenium (μg/kg) | 138.13 | 185.09 |
| Fluorine (mg/kg) |  | 1.16 |
|  |  |  |
| **Vitamins** |  |  |
| Vitamin A (iu/kg) | 3758.61 | 4628.65 |
| Vitamin D3 (iu/kg) | 2151.81 | 2822.23 |
| Vitamin E (iu/kg) | 74.09 | 94.46 |
| Vitamin B1 (mg/kg) | 5.61 | 5.73 |
| Vitamin B2 (mg/kg) | 4.86 | 5.98 |
| Vitamin B6 (mg/kg) | 6.53 | 6.7 |
| Vitamin B12 (μg/kg) | 23.49 | 28.93 |
| Vitamin C (mg/kg) | 0 | 0 |
| Vitamin K (mg/kg) | 0.68 | 0.89 |
| Folic acid (mg/kg) | 1.88 | 2.22 |
| Nicotinic acid (mg/kg) | 27.91 | 34.37 |
| Pantothetic acid (mg/kg) | 13.74 | 17.33 |
| Choline (mg/kg) | 926.94 | 1248.4 |
| Inositol (mg/kg) | 0 | 0 |
| Biotin (μg/kg) | 187.93 | 231.43 |

**Supplemental Table 2** Number of male offspring tested at 6- and 12-months of age in the PVDR task and their performance in the task during the acquisition and reversal phases.

| **Acquisition** | | | | | | | | | |
| --- | --- | --- | --- | --- | --- | --- | --- | --- | --- |
| **6 months males** | **C/C** | **C/HF** | **HF/C** | **HF/HF** | **12 months males** | **C/C** | **C/HF** | **HF/C** | **HF/HF** |
| Total animals | 24 | 37 | 28 | 27 | Total animals | 21 | 29 | 28 | 22 |
| Weight at start of testing (g, ± SD) | 25.8 ± 1.97 | 42.4 ± 4.5^Ψ^ | 26.7 ± 2.42 | 45.3 ±1.17^Φ^ | Weight at start of testing (g, ± SD) | 36.4 ± 5.64 | 48.6 ± 5.45^Ψ^ | 30.7 ± 5.18 | 48.2 ± 2.58^Φ^ |
| % reached criteria | 83.3 | 24.3* | 67.9 | 48.1 | % reached criteria | 42.9^T^ | 27.6 | 85.7* | 50.0# |
| % reversed | 66.7 | 16.2 | 67.9 | 29.6 | % reversed | 38.1 | 13.8 | 67.9 | 31.8 |
| Not reversed | 8 | 31 | 9 | 19 | Not reversed | 13 | 25 | 9 | 15 |
| - Culled due to illness | 0 | 2 | 0 | 3 | - Culled due to illness | 1 | 3 | 0 | 4 |
| - Did not reach criteria | 5 | 27 | 9 | 14 | - Did not reach criteria | 11 | 21 | 4 | 11 |
| - Failed refresh | 3 | 2 | 0 | 2 | - Failed refresh | 1 | 2 | 5 | 0 |
| **Reversal** | | | | | | | | | |
| Total animals | 16 | 6 | 19 | 9 | Total animals | 8 | 4 | 19 | 18 |
| % achieved criteria | 68.8 | 16.7 | 89.5 | 66.7 | % achieved criteria | 25.0 | 50.0 | 63.2 | 61.1 |

*p<0.013 vs C/C, #p<0.013 vs HF/C, ^T^p<0.013 vs C/C 6 months; Ψp<0.0001 vs. C/C; Φp<0.0001 vs. HF/C

**Supplemental Table 3** Number of female offspring tested at 6- and 12-months of age in the PVDR task and their performance in the task during the acquisition and reversal phases.

| **Acquisition** | | | | | | | | | |
| --- | --- | --- | --- | --- | --- | --- | --- | --- | --- |
| **6 months females** | **C/C** | **C/HF** | **HF/C** | **HF/HF** | **12 months females** | **C/C** | **C/HF** | **HF/C** | **HF/HF** |
| Total animals | 23 | 33 | 19 | 27 | Total animals | 21 | 29 | 19 | 23 |
| Weight at start of testing (g, ± SD) | 21.9± 2.11 | 41.2 ± 2.55^Ψ^ | 23.8 ± 1.75 | 40.8 ± 3.61^Φ^ | Weight at start of testing (g, ± SD) | 24.7± 2.29 | 51.8 ± 4.08^Ψ^ | 24.9 ± 1.59 | 51.4 ± 4.04^Φ^ |
| % reached criteria | 91.3 | 18.2* | 78.9 | 48.1 | % reached criteria | 85.7 | 37.9* | 78.9 | 52.2 |
| % reversed | 82.6 | 18.2 | 57.9 | 40.7 | % reversed | 57.1 | 20.7 | 57.9 | 34.8 |
| Not reversed | 4 | 27 | 8 | 16 | Not reversed | 9 | 23 | 8 | 15 |
| - Culled due to illness | 0 | 2 | 0 | 0 | - Culled due to illness | 1 | 1 | 0 | 0 |
| - Did not reach criteria | 2 | 25 | 3 | 14 | - Did not reach criteria | 2 | 18 | 4 | 11 |
| - Failed refresh | 2 | 0 | 5 | 2 | - Failed refresh | 6 | 4 | 4 | 4 |
| **Reversal** | | | | | | | | | |
| Total animals | 19 | 6 | 11 | 11 | Total animals | 8 | 6 | 11 | 8 |
| % achieved criteria | 78.9 | 50 | 90.9 | 54.5 | % achieved criteria | 25.0 | 66.7 | 90.9 | 62.5 |

*p<0.013 vs C/C; Ψp<0.0001 vs. C/C; Φp<0.0001 vs. HF/C

**Supplemental Table 4** Gene expression in 16-month-old male and female offspring relative to 10-month-old animals

|  | **10 months old** | | | | | | | |
| --- | --- | --- | --- | --- | --- | --- | --- | --- |
|  | **Males** | | | | **Females** | | | |
| **Gene** | C/C | C/HF | HF/C | HF/HF | C/C | C/HF | HF/C | HF/HF |
| *Chat* | 1.6 ± 0.7 | 1.2 ± 0.4 | 1.2 ± 0.3 | 1.2 ± 0.5 | 1.2 ± 0.3 | 1.3 ± 0.6 | 1.1 ± 0.2 | 1.3 ± 0.5 |
| *Gad1* | 1.0 ± 0.1 | 1.1 ± 0.3 | 1.2 ± 0.2 | 1.1 ± 0.2 | 1.0 ± 0.1 | 1.1 ± 0.1 | 1.0 ± 0.1 | 1.1 ± 0.2 |
| *Gad2* | 1.1 ± 0.3 | 1.3 ± 0.5 | 1.3 ± 0.4 | 1.2 ± 0.5 | 1.0 ± 0.2 | 1.2 ± 0.4 | 1.1 ± 0.3 | 1.0 ± 0.1 |
| *Htr1a* | 1.0 ± 0.04 | 1.1 ± 0.3 | 1.2 ± 0.2 | 1.1 ± 0.2 | 1.0 ± 0.1 | 1.1 ± 0.3 | 1.1 ± 0.2 | 1.0 ± 0.09 |
| *Htr2a* | 1.1 ± 0.3 | 1.1 ± 0.2 | 1.1 ± 0.1 | 1.0 ± 0.08 | 1.0 ± 0.04 | 1.0 ± 0.05 | 1.0 ± 0.03 | 1.3 ± 0.2 |
| *Slc17a7* | 1.0 ± 0.1 | 1.1 ± 0.3 | 1.1 ± 0.2 | 1.0 ± 0.2 | 1.1 ± 0.2 | 1.0 ± 0.1 | 1.0 ± 0.1 | 1.1 ± 0.2 |
|  | **16 months old** | | | | | | | |
| *Chat* | 1.3 ± 1.0 | 0.3 ± 0.3 | 1.7 ± 1.1 | 3.7 ± 2.3 | 0.4 ± 0.2 | 1.5 ± 0.5 | 0.7 ± 0.4 | 2.3 ± 1.2 |
| *Gad1* | 1.2 ± 0.4 | 0.7 ± 0.2 | 1.7 ± 0.6 | 1.6 ± 0.7 | 1.3 ± 0.3 | 1.1 ± 0.1 | 1.3 ± 0.4 | 1.5 ± 0.3 |
| *Gad2* | 0.7 ± 0.3 | 0.5 ± 0.1 | 1.1 ± 0.3 | 1.2 ± 0.6 | 0.7 ± 0.1 | 1.6 ± 0.2 | 0.9 ± 0.3 | 1.3 ± 0.5 |
| *Htr1a* | 0.8 ± 0.1 | 0.8 ± 0.09 | 1.2 ± 0.3 | 0.8 ± 0.2 | 0.7 ± 0.2 | 1.2 ± 0.1 | 0.9 ± 0.1 | 1.0 ± 0.2 |
| *Htr2a* | 1.0 ± 0.1 | 1.1 ± 0.3 | 1.0 ± 0.1 | 1.1 ± 0.2 | 0.8 ± 0.4 | 1.6 ± 0.08 | 0.8 ± 0.03 | 1.0 ± 0.08 |
| *Slc17a7* | 0.6 ± 0.1 | 0.7 ± 0.09 | 0.6 ± 0.1 | 0.4 ± 0.08* | 0.9 ± 0.2 | 0.7 ± 0.08 | 0.8 ± 0.1 | 0.6 ± 0.1 |

*p<0.05 vs. 10 months old, values from 10-month-old animals are reference
